# Tough, Self-Recoverable, Spiropyran (SP3) Bearing Polymer Beads Incorporated PAM Hydrogels with Sole Mechanochromic Behavior

**DOI:** 10.3390/gels8040208

**Published:** 2022-03-27

**Authors:** Jianxiong Xu, Yuecong Luo, Yin Chen, Ziyu Guo, Yutong Zhang, Shaowen Xie, Na Li, Lijian Xu

**Affiliations:** Hunan Key Laboratory of Biomedical Nanomaterials and Devices, College of Life Sciences and Chemistry, Hunan University of Technology, Zhuzhou 412007, China; 13658@hut.edu.cn (J.X.); luohuahua2021@163.com (Y.L.); chenyin96110@163.com (Y.C.); xiaoguo19970314@163.com (Z.G.); zyt17836227933@163.com (Y.Z.); 13502431554@163.com (S.X.); lina6980955@163.com (N.L.)

**Keywords:** mechanophore, mechanoresponsive hydrogels, emulsion polymerization, spiropyran

## Abstract

Spiropyran-containing hydrogels that can respond to external stimuli such as temperature, light, and stress have attracted extensive attention in recent years. However, most of them are generally dual or multiple stimuli-responsive to external stimuli, and the interplay of different stimulus responses is harmful to their sensitivity. Herein, spiropyran bearing polymer beads incorporated PAM (poly(AM–*co*–MA/DMSP3)) hydrogels with sole mechanochromic properties were synthesized by emulsion polymerization of acrylamide (AM) and methyl acrylate (MA) in the presence of spiropyran dimethacrylate mechanophore (DMSP3) crosslinker. Due to the hydrophobic nature of MA and DMSP3, the resultant hydrogel afforded a rosary structure with DMSP3 bearing polymer beads incorporated in the PAM network. It is found that the chemical component (e.g., AM, MA, and DMSP3 concentrations) significantly affect the mechanical and mechanoresponsive properties of the as-obtained poly(AM–*co*–MA/DMSP3) hydrogel. Under optimal conditions, poly(AM–*co*–MA/DMSP3) hydrogel displayed high mechanical properties (tensile stress of 1.91 MPa, a tensile strain of 815%, an elastic modulus of 0.67 MPa, and tearing energy of 3920 J/m^2^), and a good self-recovery feature. Owing to the mechanoresponsive of SP3, the hydrogels exhibited reversible color changes under force-induced deformation and relaxed recovery states. More impressive, the poly(AM–*co*–MA/DMSP3) hydrogel showed a linear correlation between tensile strain and chromaticity (x, y) as well as a stain and resting time-dependent color recovery rate. This kind of hydrogel is believed to have great potential in the application of outdoor strain sensors.

## 1. Introduction

In recent years, we have witnessed the prosperity of stimuli-responsive materials that can change their physical and/or chemical properties in response to external stimulation, e.g., temperature [1,2,3], pH [4], light [5], ionic strength [6,7], and magnetic/electric [8,9] fields. Such intelligent stimuli-responsive materials have shown significant potential in drug delivery [10,11,12], environmental remediation [13,14,15], artificial intelligence [16,17], wearable electronic devices [18,19,20], and so on. Inspired by the mechanical-induced deformation behavior of Mimosa in nature, the mechanoresponsive materials are particularly attractive due to their promising application in materials damage determination, human motion monitoring, and smart robots [21]. The key component of mechanoresponsive materials is the mechanophores that can change their output performance, such as electronic signals, luminescence, and appearance color under external force [22]. Usually, the detection of electronic signals and luminescence needs additional sophisticated instruments. In comparison, mechanical discoloration is intuitive visualization and is considered to be more convenient. Therefore, mechanochromic molecules have been regarded as the extremely attractive primitive in the fabrication of mechanoresponsive materials [23,24,25,26,27].

Up to now, several mechanochromic molecules, including diarylethenes, stilbenes, azobenzenes, fulgides, and spiropyrans (SP), have been reported [28]. Among them, spiropyran has received enormous attention because of its unique mechanochemistry, fast dynamic responsiveness, high fatigue resistance, and ease of functionalization [29,30,31,32,33]. Under external force-stimuli, SP can be able to undergo a reversible 6-π ring-opening reaction (spiropyran (SP) ↔ merocyanine (MC)) due to the cleavage and reformation of the C–O bond on the spiro ring [34]. During the past decades, SP mechanophores have been widely used in solid-state switches. However, the main threat in these materials is the tight molecular packing in solid states, which has significantly impeded the transformation occurring between the SP and MC forms due to the limited free volumes in the solid state. 

**Scheme 1:**
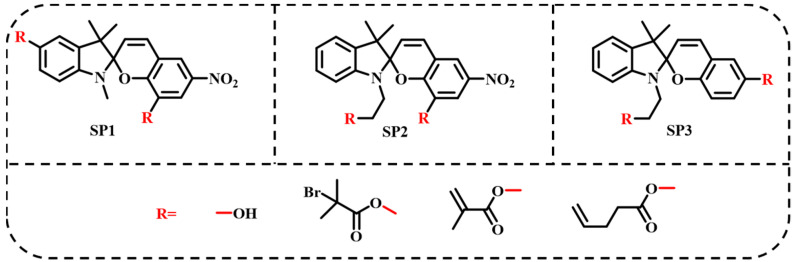
Three types of SP mechanophores distinguished from their anchoring groups for different functionalization and applications.

Consequently, the incorporation of SP into polymer networks is a valuable method to improve their stimulus responsiveness. To achieve covalent incorporation, it is necessary that the SP mechanophores contain polymerizable groups as substituents on the aromatic ring of SP. Generally, the SP mechanophores can be divided into three types (SP1, SP2, and SP3) according to the anchoring sites located at both sides of the C–O bond on the spiro ring, as shown in Scheme 1. The chemical component of SP1 and SP2 is similar. Both of them contain a nitro substituent on the aromatic ring of SP moiety. The anchoring sites of SP1 are located at the aromatic rings of each side, while the anchoring sites of SP2 are located at the aromatic ring and the nitrogen of dimethylaniline, respectively. For the SP3 mechanophore, the anchoring sites are similar to that of SP2 but lack of nitro substituent on the aromatic ring. These structural differences make them different stimulus responsiveness performances. Usually, the SP1 and SP2 mechanophores showed discoloration at the stimulus of external force, UV light, and heat, while the SP3 mechanophore only exhibited mechanochromic property [34,35,36,37,38]. Vidavsky and co-workers reported the introduction of the mechanoactive spiropyran into the polycarbonate backbone. The synthesized spiropyran–bisphenol A polycarbonate (SP–BPA–PC) was a hard glassy polymer with an elastic modulus of 1.9 GPa and a maximum elongation of only about 100% [39]. In order to further improve the ductility, Wang et al. synthesized a waterborne polyurethane polymer membrane (SP–MSPU) by grafting SP on the polyurethane chain with a maximum tensile strain of 400% and maximum tensile stress of 4 MPa [40].

Hydrogels as three-dimensional networks swollen polymers have outstanding performance (e.g., 3D porous networks, high stretchability, and excellent elastic deformation) [41,42,43,44,45]. Therefore, the fabrication of SP mechanophores encapsulated hydrogels is very important for the development of advanced strain sensors. One challenge is how to incorporate the highly hydrophobic SP mechanophores into a hydrophilic hydrogel framework. In our previous work, we have demonstrated a micelle polymerization method to encapsulate the SP1 type crosslinker into the PAM hydrogels [34]. The as-prepared SP1 containing hydrogels exhibited stimulus responsiveness under external force, UV light, and heat. However, the interplay of different stimulus responses seriously affects the response sensitivity of the hydrogel-based sensors.

Herein, a novel SP3 type crosslinker of dimethacrylate spiropyran (DMSP3) was synthesized and used for the preparation of poly(AM–*co*–MA/DMSP3) mechanically responsive hydrogels by the emulsion copolymerization of acrylamide (AM) and methyl acrylate (MA). The effect of MA:AM weight ratios and the concentration of DMSP3 crosslinker on the mechanical and mechanochromic performances were investigated to obtain the poly(AM–*co*–MA/DMSP3) hydrogels with excellent mechanical properties. Moreover, the stimulus responsiveness and self-recovery ability were also tested. Ascribed to the presence of SP3, the hydrogel exhibited reversibility of discoloration in the stretched and original state. The discoloration behavior was only responded to a single mechanical force stimulation and not disturbed by ultraviolet light and thermal stimulation, which ensured the accuracy of the sensing signal. Furthermore, mechanochromic properties were further researched by building a quantitative relationship between the external force stimulation and color change and evaluating the reversible color recovery times.

## 2. Results and Discussion

**Figure 1:**
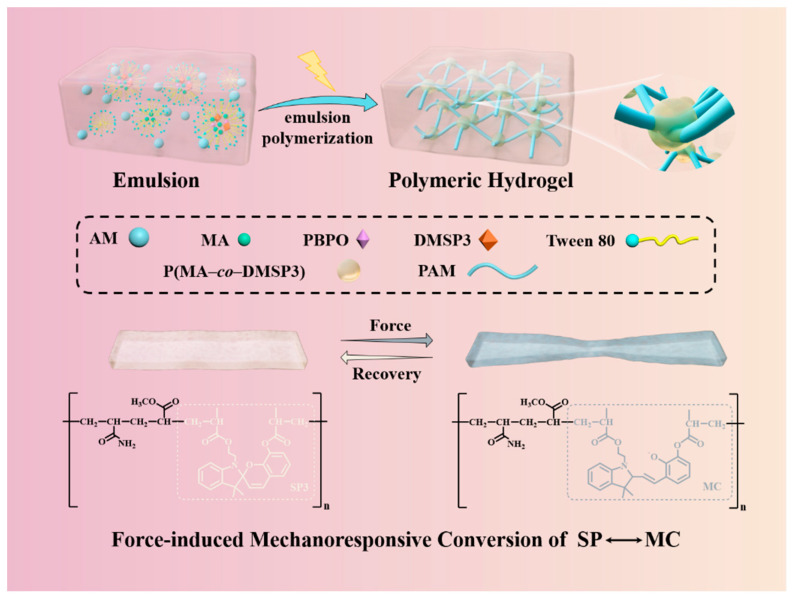
Scheme for the preparation of poly(AM–*co*–MA/DMSP3) hydrogels and the mechanism of their mechanochromic characteristic.

The tough, self-recoverable, spiropyran bearing polymer beads incorporated poly(AM–*co*–MA/DMSP3) hydrogels were synthesized via photo-initiated emulsion polymerization. The preparing process was schematically shown in Figure 1. Initially, hydrophobic MA monomer, DMSP3 crosslinker, and PBPO photo-initiator were homo-dispersed in the aqueous solution of AM monomer with the help of TWEEN 80 to form a stable emulsion. In this state, the hydrophobic species mainly existed in surfactant stabilized oil droplets and surfactant micelles. Once the polymerization was triggered by photo-irradiation, well-dispersed spiropyran bearing polymer beads (P(MA/DMSP3)) was formed. With the progress of the polymerization, hydrophilic PAM chains were produced and covalently attached on the surface of the P(MA/DMSP3) bead due to the copolymerization of AM and MA. After the completion of the polymerization, rosary-like three-dimensional poly(AM–*co*–MA/DMSP3) polymer hydrogels with soft PAM as “threads” and hard P(MA/DMSP3) polymer microspheres as “beads” were obtained. Due to the unique structure and the presence of DMSP3 mechanochromic probes, the poly(AM–*co*–MA/DMSP3) hydrogels were expected to have excellent mechanical properties and mechanochromic characteristics. 

**Figure 2:**
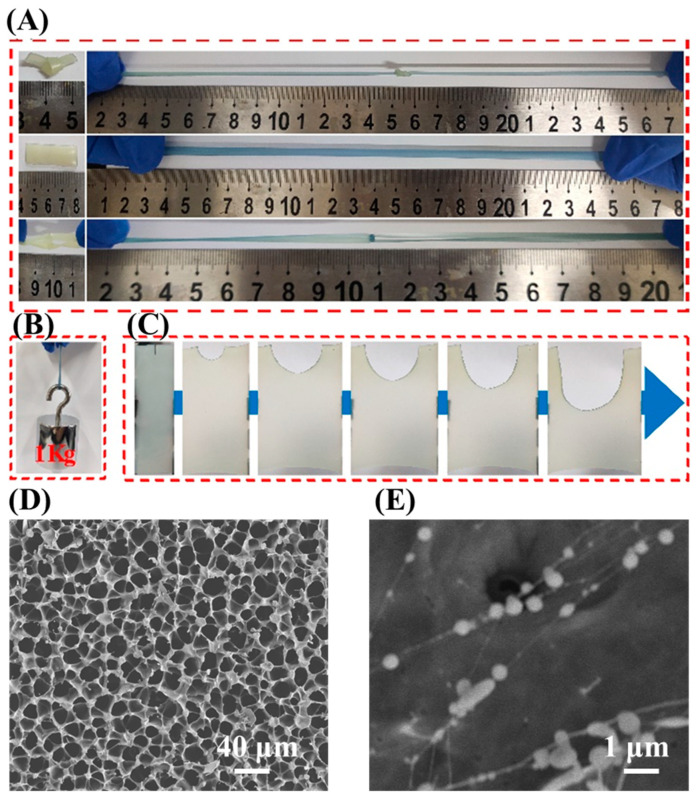
Visual photograph of the mechanical and mechanoresponsive properties of as-prepared poly(AM–*co*–MA/DMSP3) hydrogels by (**A**) knotted, original, and crossover stretching; (**B**) holding 1 kg of weight; (**C**) resisting crack propagation, (**D**,**E**) SEM images of poly(AM–*co*–MA/DMSP3) hydrogels with micellar structures at different magnifications.

Herein, we choose the AM_25_–DMSP3_0.4_–MA_25_ hydrogels (the preparation condition can be found in Table 1) as a typical example to check their structure and properties. AM_25_–DMSP3_0.4_–MA_25_ hydrogels displayed extraordinary mechanical and flexible properties. As shown in Figure 2A, the hydrogels can withstand knotted stretching, original stretching, and crossover stretching up to six times their original length without any observable damage. In parallel, the hydrogels can bear up to 1 kg of weight, which is about 1000 times their own weight (Figure 2B). Figure 2C displays the crack propagation process of the AM_25_–DMSP3_0.4_–MA_25_ hydrogels with a cut notch (~5 mm). The result showed that with the increase in tensile strain (λ), the notch was obviously passivated and gradually developed into a semicircular crack, indicating the excellent toughness of the hydrogels. It was interesting to note that in all stretching states, a pronounced color change of the hydrogels from light yellow to blue-gray was observed in areas of stress concentration and deformation, confirming the mechanochromic property of the hydrogels. The mechanochromic mechanism of the hydrogels was due to the mechanical activation of SP-to-MC transition in the polymer network, as schematically shown in Figure 1. The inner structure of the hydrogels was observed by SEM. As shown in the SEM image (Figure 2D), the AM_25_–DMSP3_0.4_–MA_25_ hydrogels presented as a porous honeycomb structure. At the SEM image of high magnification (Figure 2E), it can be seen that polymer beads with an average size of 200~500 nm were uniformly embedded in the framework of the hydrogels. We called this structure a three-dimensional rosary interpenetrating polymer network.

**Figure 3:**
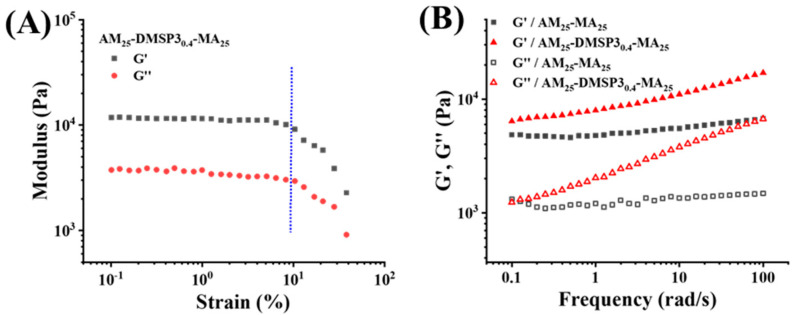
(**A**) The strain amplitude sweep test of AM_25_–DMSP3_0.4_–MA_25_ hydrogel at a fixed angular frequency (10 rad/s) at 25 °C; (**B**) Variation of storage modulus G’ and loss modulus G″ as a function of frequency for AM_25_–MA_25_ hydrogels and AM_25_–DMSP3_0.4_–MA_25_ hydrogels measured at λ = 1%.

The rheological property of the hydrogels was evaluated. Figure 3A showed the strain amplitude sweep test of AM_25_–DMSP3_0.4_–MA_25_ hydrogels at a fixed angular frequency (10 rad/s) at 25 °C. As shown, the storage modulus G’ and loss modulus G″ are independent of the applied strain at lower strain (λ = 0~10%). Moreover, the G’ is always larger than the G″. The results suggested that the AM_25_–DMSP3_0.4_–MA_25_ hydrogels exhibited a typical elastic response with a linear viscoelastic region at λ = 0~10%. The rheological property of A AM_25_–DMSP3_0.4_–MA_25_ and AM_25_–MA_25_ hydrogels were compared by checking the G’ and G″ variation as a function of frequency at a fixed strain of λ = 1% (Figure 3B). It can be observed that the G’ and G″ of AM_25_–DMSP3_0.4_–MA_25_ hydrogels were larger than those of AM_25_–MA_25_ hydrogels in all frequency ranges, implying that the addition of DMSP3 significantly improved the viscosity and elasticity of the hydrogel. The results also demonstrated the crosslinked structure of the AM_25_–DMSP3_0.4_–MA_25_ hydrogels.

**Figure 4:**
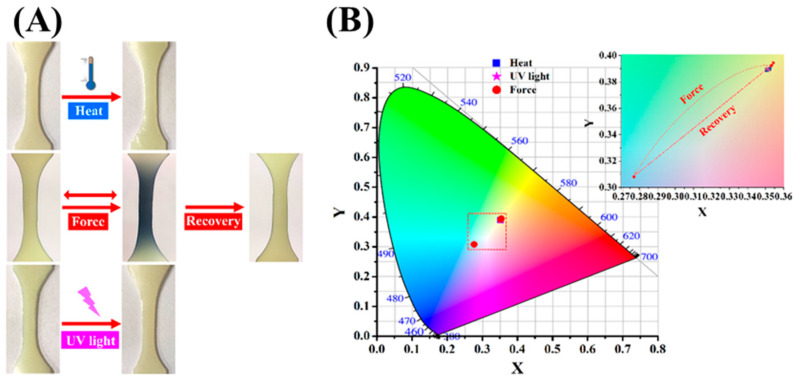
(**A**) Optical images of poly(AM–*co*–MA/DMSP3) hydrogels under the stimuli of heat, external force (λ = 3), and UV light, and (**B**) their corresponding CIE color coordinates.

Apart from mechanoresponsive property, we also investigate the photochromic and thermochromic properties of the AM_25_–DMSP3_0.4_–MA_25_ hydrogels. As shown in Figure 4A, the as-prepared AM_25_–DMSP3_0.4_–MA_25_ hydrogels were exposed to a UV light irradiation (365 nm) for 10 min and heated at 60 ℃ for 10 min. Optical images were taken before and after UV exposure and heating. Obviously, visual inspection of AM_25_–DMSP3_0.4_–MA_25_ hydrogels showed no color change after heating and UV light irradiation, indicating no SP-to-MC transition in the gel networks. This result confirmed that the DMSP3 mechanophore had no photochromism and thermochromism, which might be due to the absence of the electron-withdrawing nitro group at the 6-position of the benzopyran. Under external force stimuli (λ = 3), the as-prepared AM_25_–DMSP3_0.4_–MA_25_ hydrogels exhibited a color change from light yellow to blue-gray color. Moreover, the blue-gray color gradually faded and returned to the initial light yellow color in approximately 30 min after removing the external force, suggesting the reversible mechanoresponsive property of the AM_25_–DMSP3_0.4_–MA_25_ hydrogels. To quantitatively observe the color-changing degree, the gauge section of digital images of the gels were analyzed by the RGB (red, green, blue) values and located in the x, y chromaticity diagram (CIE 1931 color space). As shown in Figure 4B, during the deformation process, the gels showed an obvious color change in the pathways towards the blue-gray color under the stimuli of force and returned to the initial light yellow color without external force, whereas the colors of the hydrogels before and after UV light and temperature stimuli were located in similar light yellow areas. The results demonstrated the sole reversible mechanoresponsive property of the hydrogels.

**Figure 5:**
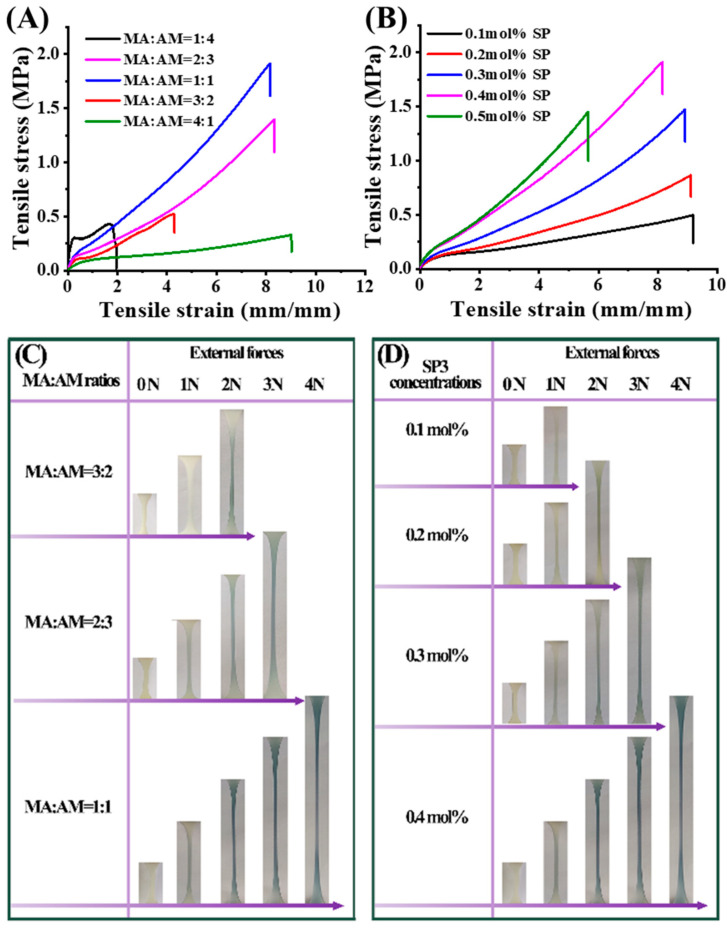
Mechanical and mechanoresponsive properties of the as-prepared poly(AM–*co*–MA/DMSP3) hydrogels with different MA:AM ratios and DMSP3 concentrations: (**A**,**B**) tensile stress–strain tests; (**C**,**D**) their corresponding color change in response to the external force.

In order to obtain poly(AM–*co*–MA/DMSP3) hydrogels with superior properties, the effect of MA:AM weight ratios and the concentration of DMSP3 crosslinker on the mechanical and mechanochromic performances were evaluated. To this end, a series of poly(AM–*co*–MA/DMSP3) hydrogels were firstly prepared under different weight ratios of MA:AM. The concentration of the DMSP3 crosslinker was kept at a constant value of 0.4% in proportion to the molar amount of MA monomer (0.4 mol%). The preparation conditions are summarized in Table 1. Figure 5A illustrates the typical stress–strain curves of poly(AM–*co*–MA/DMSP3) hydrogels with different MA:AM weight ratios. Obviously, at the MA:AM ratio of 1:4, the AM_25_–DMSP3_0.4_–MA_40_ hydrogels showed weak tensile stress (σ) of 0.32 MPa at a large tensile strain (λ) of ~900%. Generally, the tensile stress increased with the increasing of the weight ratio of MA:AM from 1:4 to 1:1. However, when the MA:AM ratio was beyond 1:1, the poly(AM–*co*–MA/DMSP3) hydrogels became brittle, accompanied by a significant decrease in tensile stress and tensile strain. Further, the effect of DMSP3 crosslinker concentration on the mechanical properties of the hydrogels at a fixed weight ratio of MA:AM at 1:1 was examined. Similarly, poly(AM–*co*–MA/DMSP3) hydrogel showed different mechanical properties with different DMSP3 concentrations (Figure 5B). As the DMSP3 concentrations increased from 0.1 mol% to 0.4 mol%, the hydrogels showed a monotonical increase in tensile stress from 0.49 to 1.91 MPa and elastic modulus from 0.27 to 0.67 kPa at similar fracture strains of ~800%. When the DMSP3 concentration further increased to 0.5 mol%, the poly(AM–*co*–MA/DMSP3) hydrogel exhibited enhanced stiffness, resulting in an inferior mechanical strength. Based on the above results, the AM_25_–DMSP3_0.4_–MA_25_ hydrogel prepared with the weight ratio of MA:AM at 1:1 and DMSP3 concentration at 0.4 mol% achieved the most remarkable mechanical properties (σ of 1.91 MPa, λ of 815%, and elastic modulus (E) of 0.67 kPa). In parallel, we also comparatively observed the external force-dependent color change of poly(AM–*co*–MA/DMSP3) hydrogels using tensile tests. Figure 5C,D summarized the color change of the hydrogels in response to an external force. As revealed, the AM_25_–DMSP3_0.4_–MA_25_ hydrogel strips under larger external force and the degree of discoloration in the blue-gray direction was greater. Thus, the AM_25_–DMSP3_0.4_–MA_25_ hydrogels were chosen as the research object in the following discussion, if specialty pointed out otherwise.

**Figure 6:**
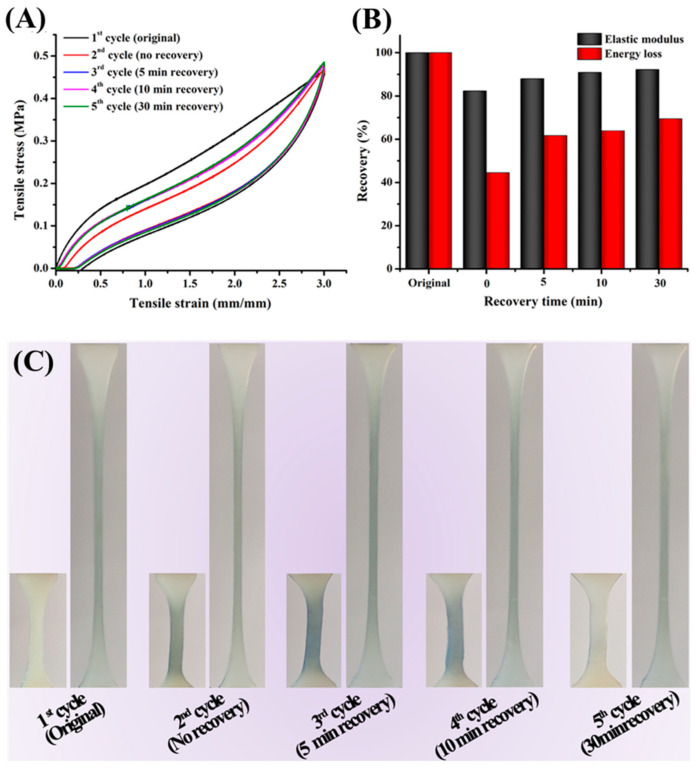
Force-induced color change and mechanical recovery of AM_25_–DMSP3_0.4_–MA_25_ hydrogels using cyclic loading–unloading tests, as demonstrated by (**A**) hysteresis loading–unloading tests; and (**B**) toughness (energy loss) and stiffness (elastic modulus) recovery; (**C**) visual inspection of color change and recovery.

Due to the elastomer-like mechanical property and reversible transition of SP ↔ MC in DMSP3 moiety, the AM_25_–DMSP3_0.4_–MA_25_ hydrogels were expected to have mechanical and mechanoresponsive self-recovery properties. To demonstrate the self-recovery properties of the hydrogels, loading and unloading experiments were performed on the AM_25_–DMSP3_0.4_–MA_25_ hydrogels with a maximum tensile strain of λ = 3. For comparison, the first two loading–unloading tests (i.e., first original and second no recovery) were carried out continuously without any rest period, while the third–fifth tests (i.e., third, fourth, and fifth-recovery) were conducted on the gels with 5, 10 and 30 min, respectively, for recovery during the unloading process. For each loading–unloading cycle, the hydrogel strip was spontaneously recovered to its original length without additional treatment. The mechanical recovery was estimated by cyclic stress–strain curves (Figure 6A), and the stiffness/toughness recovery ratios were summarized in Figure 6B. As shown in Figure 6A, the AM_25_–DMSP3_0.4_–MA_25_ hydrogels showed the largest hysteresis loop in the first original cycle, and the hysteresis loop became much smaller in the second no recovery cycle. Nevertheless, the hysteresis loop became larger with increasing the resting time in the third–fifth cycles. Quantitatively, the stiffness/toughness recovery of hydrogels after the second no recovery cycle was 82.0%/43.4%. After recovery for 5 min in the third cycle, the stiffness/toughness recovery of hydrogels increased to 88.0%/61.7%. For fourth and fifth loading–unloading cycles, with the resting time increased to 10 and 30 min, respectively, the stiffness/toughness recovery of hydrogels reached 91.0%/63.8% and 92.2%/69.5%, respectively. The results suggested that prolonged resting time benefited the mechanical recovery of the hydrogels. During the loading–uploading test, the mechanoresponsive self-recovery ability of the hydrogels was also evaluated by observing the color changes of the hydrogel strips at each loading–unloading cycle. As shown in Figure 6C, the color of the AM_25_–DMSP3_0.4_–MA_25_ hydrogel strip was changed from light yellow to blue-gray at the stress-concentrated area when the length of the hydrogel becomes three times the original length. Without any resting time, the hydrogels immediately self-recovered to their original length but still displayed blue-gray color at the beginning of the second cycle. Similarly, the hydrogels were also in blue-gray color at the beginning of the third and fourth cycles after a 5 or 10 min resting time, respectively. Notably, the hydrogels recovered to their original light yellow color after 30 min resting time at the fifth cycle, suggesting that prolonged resting time was favorable for the reversion of MC-to-SP.

**Figure 7:**
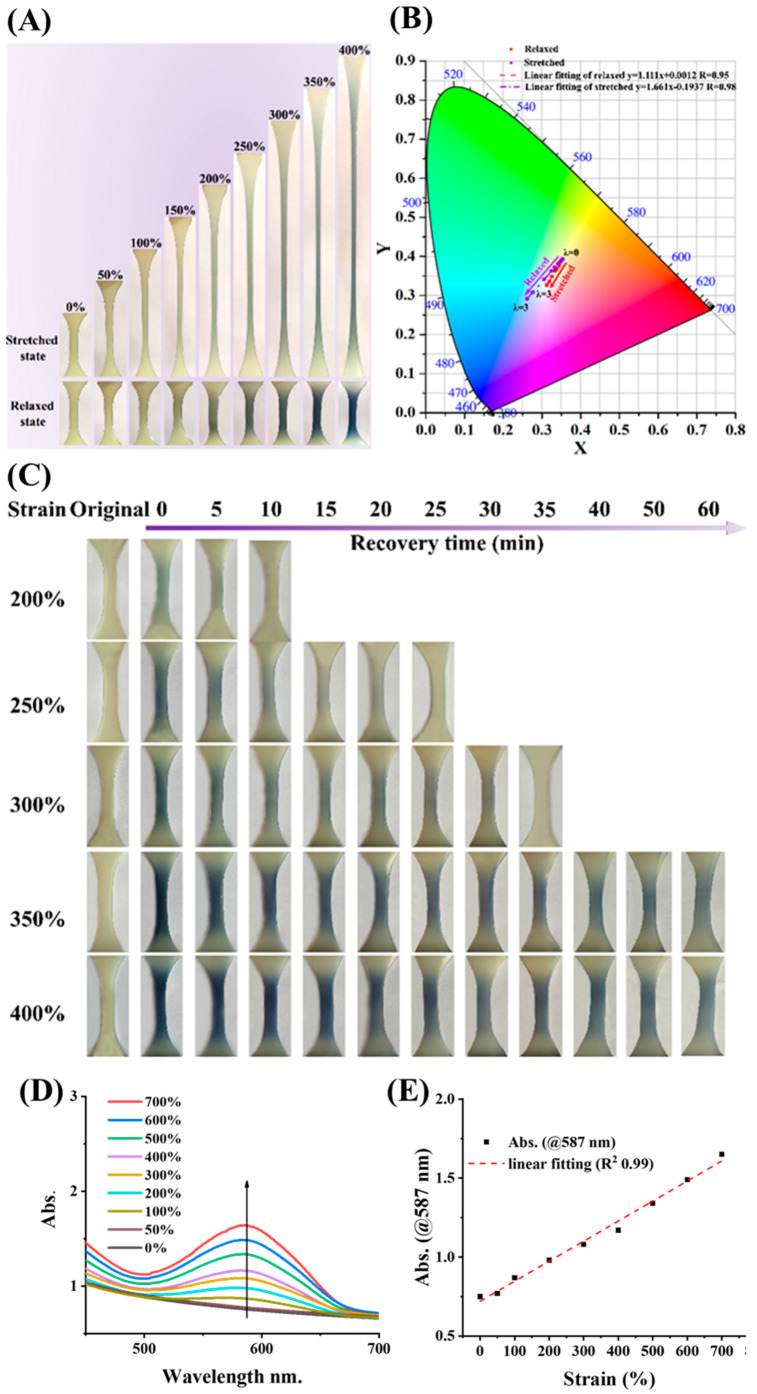
(**A**) Optical images of the corresponding color changes of AM_25_–DMSP3_0.4_–MA_25_ hydrogels under continuous loading and unloading tests with different strains, (**B**) their corresponding CIE color coordinates, (**C**) Color recovery of poly(AM–*co*–MA/DMSP3) hydrogel after as a function of recovery time under daylight and room temperature, (**D**) UV-vis spectrum of poly(AM–*co*–MA/DMSP3) hydrogels at different strains of 0–700%, (**E**) Data fitting to UV-vis absorbance at 587 nm for the gels at different strains of 0–700%.

In order to attain deep insight into the mechanochromic property, a quantitative relationship between the external force stimulation and color change was evaluated by successive loading–unloading tests conducted on AM_25_–DMSP3_0.4_–MA_25_ hydrogels at different strains, and the corresponding color changes were recorded by optical images. As shown in Figure 7A, the as-prepared gels did not show color changes at stretched or relaxed states when the tensile strain was less than 150%. This suggested that the small stretching force was dissipated to endure the deformation of the PAM network and was not large enough to be transferred from the PAM network to P(MA/DMSP3) polymer beads to trigger the SP to MC transformation or the transition degree of SP → MC was not large enough to induce the color change being observed by naked eyes. Obvious color changes can be observed as the tensile strain was larger than 200%, and the blue-gray coloration gradually deepened with the increase in tensile strains since a large tensile strain was beneficial for the conversion of SP to MC. Similarly, the relaxed gel can be recovered immediately to its original length immediately but still retain blue-gray color at each unloading state. To quantitatively analyze the color changes in the gels, the optical color change of the stress-concentrated area of the gels between the stretched and relaxed states were monitored using the RGB (red, green, blue) color channels and further located into the x, y chromaticity diagram. As shown in Figure 7B, a linear color change path of the stretched gels from light yellow to blue-gray with a linear fitting R^2^ of 0.98 was observed as the tensile strain increased from 0% to 400%. In parallel, the relaxed gels showed a distinct pathway toward blue-gray color after unloading, with a linear fitting R^2^ of 0.95. This result provided strong evidence that the gel indeed displayed strain-dependent color changes. The different color change pathways during the stretching and relaxing states may be due to the secondary color change by the isomerization and accumulation of MC. We also use UV–vis spectrum to quantitatively evaluate the mechanical activation of SP-to-MC transition degree of the hydrogels under different strains since the SP and MC moieties have distinguishable UV absorptions. Figure 7D showed the UV–vis spectra of the hydrogels at different strains of 0–700%. As shown, the hydrogel at a low strain of 50% showed almost identical UV–vis spectra to the virgin gel, suggesting that the SP → MC conversion of the gels cannot be detected under the low strains of 0% to 50%. When the gels were stretched to 100% or above, a new adsorption peak located at 587 nm corresponding to the MC moiety was observed, and the peak intensity increased with the increase of strains. The discoloration threshold of the hydrogels is about 100%. The peak intensity as a function of strains was summarized in Figure 7E. A linear relationship with an R^2^ value of 0.99 was obtained, further confirming that the SP → MC conversion rate was related to the strains. Additionally, we also examined the recovery times on the same gel samples after being stretched at the different tensile strains from 200% to 400%. As shown in Figure 7C, the relaxed gels can be recovered to the original light yellow color gradually by prolonging the resting time. Moreover, the relaxed gels needed more time to recover from blue-gray to light yellow after stretching at larger tensile stress. For example, 10 min was needed for the gels to be recovered to their original light yellow color after stretching at λ = 2, 25 min was needed for λ = 2.5, 35 min was needed for λ = 3, and more than 60 min was needed for λ larger than 3.5. Based on the linear correlation between strain and chromaticity (x, y) as well as the self-recovery ability, it is expected that the hydrogels can be applied to strain sensors by monitoring the corresponding color change or color change path of the hydrogels due to different strains and vice versa. Furthermore, the reversible and mechanoresponsive property of poly(AM–*co*–MA/DMSP3) hydrogels can be instantly used for rewritable printing and rewritable data storage.

## 3. Conclusions

In summary, a novel SP3 type crosslinker of dimethacrylate spiropyran (DMSP3) was synthesized and introduced into the copolymer of acrylamide (AM) and methyl acrylate (MA) by emulsion polymerization to prepare poly(AM–*co*–MA/DMSP3) mechanically responsive hydrogel. Under optimized conditions, the prepared poly(AM–*co*–MA/DMSP3) hydrogel presented as a rosary-like three-dimensional network structure with SP3 bearing polymer beads incorporated in PAM frameworks. Moreover, the hydrogels exhibited excellent mechanical properties (tensile stress of 1.91 MPa, a tensile strain of 815%, an elastic modulus of 0.67 MPa, and tear energy of 3920 J/m^2^). In addition, the obtained poly(AM–*co*–MA/DMSP3) hydrogel only responded to a single mechanical force stimulus, and the response signal was not disturbed by the stimulation of heat and ultraviolet light, which ensured response sensitivity of the hydrogel-based sensors. The hydrogel showed a transition from light yellow to blue-gray under external stimulation, and when the external stimulation was removed, it could return to its original color in a short time, and the color change was reversible. More impressive, the poly(AM–*co*–MA/DMSP3) hydrogel showed a linear correlation between strain and chromaticity (x, y) as well as a stain and resting time-dependent color recovery rate. Based on this, poly(AM–*co*–MA/DMSP3) hydrogels are expected to serve as a mechanically responsive sensor for direct, simple, and visual detection of material damage/sensing/imaging.

## 4. Materials and Methods

### 4.1. Materials

Acrylamide (AM, 99%), methyl acrylate (MA, 99%), ethylene glycol dimethacrylate (98%), phenylbis(2,4,6-trimethylbenzoyl)phosphine oxide (PBPO), TWEEN 80, *o*-vanillin, boron tribromide (BBr_3_), 2-iodoethanol, 2,3,3-trimethyl-3H-indole, and methacrylic anhydride were purchased from Shanghai Aladdin Chemistry Co., Ltd. (Shanghai, China). All chemical reagents were used directly as received without further purification. Water used in this work was purified by a DI-RO water purification system.

### 4.2. Synthesis of Dimethacrylate Spiropyran Mechanophore (DMSP3) Crosslinker

**Scheme 2:**
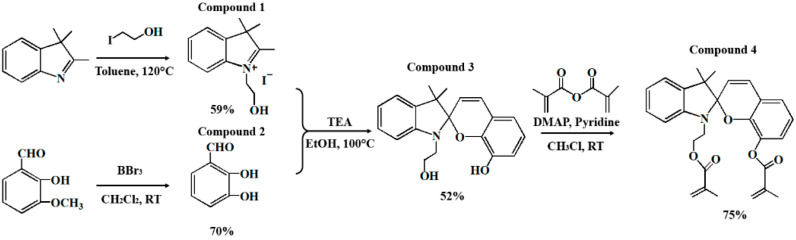
Scheme of the stepwise preparation of DMSP3 crosslinker.

The DMSP3 crosslinker (compound **4**) was synthesized in four steps from compound **1** (2-hydroxyethyl-2,3,3-trimethyl-3H-indolium iodide) and compound **2** (2,3-dihydroxybenzaldehyde) in the presence of triethylamine and further functionalized with methylacryloyl ester group by reaction with methacrylic anhydride. Compound **1** was synthesized by the alkylation reactions of 2,3,3-trimethyl-3*H*-indole and 2-iodoethanol to give iodide salt (2-hydroxyethyl-2,3,3-trimethyl-3H-indolium iodide). Compound **2** was synthesized by replacing the methoxy group of *o*-vanillin with a hydroxyl group by hydrolysis reaction with BBr_3_ to obtain 2,3-dihydroxybenzaldehyde. The reaction equations were schematically shown in Scheme 2. The chemical structure of all the intermediates and the final DMSP3 crosslinker was con-firmed by 1H NMR as shown in Figures S1–S4.

Compound **1** (2-hydroxyethyl-2,3,3-trimethyl-3H-indolium iodide). To a 500 mL round-bottom flask equipped with a reflux condenser, 150 mL acetonitrile, 2,3,3-trimethyl-3*H*-indole (11.2 g, 70 mmol, 1 equiv), and 2-iodoethanol (6.5 mL, 84 mmol, 1.2 equiv) were sequentially added. Then, the reaction system was heated up to 85 °C and reacted for 12 h. After the completion of the reaction, the mixture was cooled down to ambient temperature. The solvent of acetonitrile was distilled off under reduced pressure. The resulting precipitate was washed with CHCl_3_ three-time (3 × 50 mL) and dried under vacuum (0.1b kpa, 60 ℃) overnight, finally obtaining a light purple solid powder compound **1** (13.7 g, 41 mmol 59% yield). ^1^H NMR (300 MHz, DMSO-d_6_) δ 7.96–7.93 (m, ^1^H), 7.86–7.83 (m, 1H), 7.62–7.59 (m, ^2^H), 4.60–4.57 (t, ^2^H), 3.88−3.85 (t, ^2^H), 2.81 (s, ^3^H), 1.54 (s, ^6^H). 

Compound **2** (2,3-dihydroxybenzaldehyde). To a 500 mL round-bottom flask equipped with a dropping funnel, 250 mL CH_2_Cl_2_ and *o*-vanillin (15.0 g, 98.5 mmol, 1 equiv) were added. The reaction system was placed in an ice bath and cooled to 0 °C. After that, 30 mL CH_2_Cl_2_ contained BBr_3_ (31.8 g, 126.9 mmol, 1.3 equivalent) in the dropping funnel was added dropwise to the reaction solution within 30 min under stirring. The mixture solution was stirred and reacted at room temperature for 19 h. After the reaction, 100 mL of water was added, and the mixture solution was further stirred for 1 h. The solution was then extracted with EtOAc (3 × 100 mL) and washed with saturated brine (3 × 100 mL) for further purification. The collected solution was dried with anhydrous Na_2_SO_4_ overnight and concentrated in vacuo to give a dark purple residue. The crude product was recrystallized by hot hexanes (50 °C) and acquired a yellow crystal compound **2** (10.9 g, 79 mmol, 70% yield). ^1^H NMR (300 MHz, CDCl_3_) δ 11.09 (s, ^1^H), 9.90 (s, ^1^H), 7.20–7.14 (m, ^2^H), 6.97–6.92 (m, ^1^H), 5.61(s, ^1^H).

Compound **3** (dihydroxyl spiropyran). To a 500 mL round-bottom flask, compound 1 (13.9 g, 42 mmol, 1.05 equivalent), compound **2** (5.5 g, 40 mmol, 1 equivalent), and triethylamine (6.4 mL, 80 mmol, 2 equivalent) were soluted in 150 mL ethanol. The reaction mixture was brought to reflux under N_2_ pressure and was stirred for 10 h. After cooling down to ambient temperature, the precipitate was filtered out and washed with cooled ethanol (3 × 20 mL) to yield compound **3** as a dark purple solid powder (6.9 g, 21 mmol, 52%). ^1^H NMR (300 MHz, CDCl_3_) δ 7.13–6.61 (m, 8H), 6.26 (s, ^1^H), 6.11 (s, ^1^H), 5.87–5.85 (d, ^1^H), 5.57 (s, ^1^H), 5.34 (s, ^1^H), 4.29–4.26 (m, ^2^H), 3.43–3.38 (m, ^2^H), 1.97 (s, ^3^H), 1.93 (s, ^3^H), 1.31 (s, ^3^H), 1.16 (s, ^3^H).

Compound **4** (dimethacrylate spiropyran (DMSP3)). To a 250 mL round-bottom flask, compound **3** (6.4 g, 20 mmol, 1 equivalent) and *N*,*N*-dimethylaminopyridine (4.8 g, 40 mmol, 2 equivalent) was dissolved in 100 mL dry CH_2_Cl_2_. The reaction mixture was cooled to 0 °C and 20 mL CH_2_Cl_2_ contained methacrylic anhydride (8.9 mL, 60 mmol, 3 equivalent) were added dropwise to the cold solution under N_2_ pressure within 15 min. The reaction system was stirred overnight under ambient temperature. After the reaction finished, the mixture solution was washed with 1 M HCl (2 × 100 mL) and saturated brine (2 × 100 mL) for further purification. The collected solution was dried by anhydrous MgSO_4_ overnight and concentrated by rotary evaporation to give a gray solid. The crude product was purified by column chromatography eluting with 0.5% Et_3_N/CH_2_Cl_2_ to yield DMSP3 as light purple oil (6.8 g, 15 mmol, 75%). ^1^H NMR (300 MHz, CDCl_3_) δ 7.13–6.61 (m, ^8^H), 6.26 (s, ^1^H), 6.11 (s, ^1^H), 5.87–5.78 (m, ^2^H), 5.57 (s, ^1^H), 4.29–4.26 (m, ^2^H), 3.43–3.38 (m, ^2^H), 1.97 (s, ^3^H), 1.93 (s, ^3^H), 1.31 (s, ^3^H), 1.16 (s, ^3^H).

### 4.3. Synthesis of Poly(AM–co–MA/DMSP3) Hydrogels 

Briefly, hydrophobic monomer of MA (2.5 g, about 25 wt% of total mass) and DMSP3 crosslinker (0.0532 g, 0.4 mol% of MA), and photo-initiator PBPO (0.0365 g, 0.3 mol% of MA) were added into 1 wt% TWEEN 80 aqueous solution (5 g, about 50 wt% of total mass) and vortexed for 5 min to form a uniform emulsion. Then, the hydrophilic monomer of AM (2.5 g, 25 wt% of total mass) monomer was added to the emulsion and vortexed for 5 min to dissolved into the aqueous phase of the emulsion. The mixture solution was injected into a glass mold with a 1 mm thick Teflon spacer and exposed to white light for 1 h to form poly(AM–*co*–MA/DMSP3) hydrogels. All poly(AM–*co*–MA/DMSP3) hydrogels were prepared in the same way just by tailoring the contents of MA, AM, and DMSP3 crosslinker in the gels. To further distinguish different poly(AM–*co*–MA/DMSP3) hydrogels were named as AM_X_–SP_Y_–MA_Z_, where X, Y, and Z represented the contents of AM, DMSP3, and MA in the gels, respectively. For example, the above-prepared poly(AM–*co*–MA/DMSP3) hydrogels can be named AM_25_–DMSP3_0.4_–MA_25_. The AM_25_–MA_25_ hydrogels were prepared in the same way as the AM_25_–DMSP3_0.4_–MA_25_ hydrogels described above, except for the addition of the DMSP3 crosslinker.

### 4.4. Mechanical Tests

The hydrogels were prepared in a 1 mm thickness mold and cut into a standard dumbbell shape (ASTM-638-V) with 3.18 mm in width and 25 mm in gauge length before the tests. The tensile strain (λ), tensile stress (σ), elastic modulus (*E*), and dissipated energy (*U_hys_*) were measured using a tensile tester (UTM 4304, SUNS) equipped with a 100 N load cell. The crosshead speed during the test was fixed at 100 mm·min^-1^. The tensile strain was calculated by the elongation of the sample (Δ*l*) to its initial length (*l*_0_) (λ = Δ*l*/*l*_0_). The tensile stress (σ) was defined as the load force (*F*) applied per unit of the original cross-sectional area (A_0_) of the sample (σ = *F*/A_0_). The elastic modulus (*E*) was calculated by the slope of the initial linear regime of the stress-strain curve. The dissipated energy (*U_hys_*) was estimated between the loading and unloading cycles. Before the tearing energy test, the specimen was cut into a trouser shape of 40 mm in length and 20 mm in width. The tearing energy (*T*) was calculated by the average force (*F_ave_*) during steady-state tearing to the width (*w*) of the specimen (*T* = 2*F_ave_*/*w*).

### 4.5. Rheological Measurement

The rheological properties of the prepared hydrogels were measured on a TA 2000ex rheometer using plate-and plate geometry (diameter 25 mm, gap 1000 μm), through two different modes:(i) the dynamic strain sweep from 0.1~1000% with a constant frequency of 10 rad/s was first performed at 25 °C, and the storage modulus was recorded to define the linear viscoelastic region in which the storage modulus is independent of the strain amplitude; (ii) the viscoelastic parameters, including shear storage modulus and loss modulus were measured over the ω range of 0.1–100 rad/s at strain 1% at 25 °C.

### 4.6. Optical Color Characterization

A white board was applied to the background during the test under ambient room light conditions. All-Optical images were taken by a Nikon D7000 camera. To further balance the white background, the background of all images was split into RGB channels and obtained the RGB value of pure white (RGB values of 220, 220, and 220) through Image-J software. After the background was white-balanced, the RGB values of the gel specimen center were obtained from the histogram of the area. To directly identify the color change of the gel specimen in response to force, the RGB values were converted to the (x, y) value and marked in the chromaticity diagram (CIE 1931 color space).

### 4.7. UV-Vis Spectrometer

The gel specimens of poly(AM–*co*–MA/DMSP3) hydrogel with the size of 26 mm × 9 mm × 1 mm were placed on the wall of 1 cm path length quartz cuvette. The UV-Vis spectra of gel specimen after being stretched to various strains were obtained using a UV-Visible spectrophotometer (TU-1810, PERSEE) over the 300−800 nm wavelength range.

## Figures and Tables

**Table 1:** Synthetic formula and mechanical properties of the poly(AM–*co*–MA/DMSP3) hydrogels prepared under different conditions.

poly(AM–*co*–MA/SP3) Hydrogels	Water Contents (wt%)	MA:AM Ratio	MA–SP3–AM Concentrations (mol%)	σ (MPa)	λ (mm/mm)	*E* (KPa)
MA_10_-SP3_0.4_-AM_40_	50	1:4	0.4	0.43	1.64	1.45
MA_20_-SP3_0.4_-AM_30_	50	2:3	0.4	0.52	4.29	0.57
MA_25_-SP3_0.4_-AM_25_	50	1:1	0.4	1.91	8.15	0.67
MA_30_-SP3_0.4_-AM_20_	50	3:2	0.4	1.39	8.30	0.58
MA_40_-SP3_0.4_-AM_10_	50	4:2	0.4	0.32	8.90	0.20
MA_25_-SP3_0.1_-AM_25_	50	1:1	0.1	0.49	9.13	0.27
MA_25_-SP3_0.2_-AM_25_	50	1:1	0.2	0.85	9.04	0.28
MA_25_-SP3_0.3_-AM_25_	50	1:1	0.3	1.47	9.90	0.39
MA_25_-SP3_0.5_-AM_25_	50	1:1	0.5	1.45	5.64	0.71

## Data Availability

Not applicable.

## Supplementary Materials

The following are available online at https://www.mdpi.com/article/10.3390/gels8040208/s1, Figure S1: 1H NMR spectrum of 2-hydroxyethyl-2,3,3-trimethyl-3H-indolium iodide. Figure S2: 1H NMR spectrum of 2,3-dihydroxybenzaldehyde. Figure S3: 1H NMR spectrum of dihydroxyl spiropyran. Figure S4: 1H NMR spectrum of DMSP3 crosslinker.
