# Supplementary material for: Tough, Self-Recoverable, Spiropyran (SP3) Bearing Polymer Beads Incorporated PAM Hydrogels with Sole Mechanochromic Behavior
Source: Gels. 2022 Mar 27;8(4):208. doi: 10.3390/gels8040208 (PMC9031960; doi:10.3390/gels8040208)
Supplement: Supplementary file 1 [file gels-08-00208-s001.zip › gels-1636329-supplementary.pdf]

gels

MDPI

Supplementary Materials

Tough, Self-Recoverable, Spiropyran (SP3) Bearing Polymer Beads Incorporated PAM Hydrogels with Sole Mechanochromic Behavior

Jianxiong Xu, Yuecong Luo, Yin Chen, Ziyu Guo, Yutong Zhang, Shaowen Xie, Na Li and Lijian Xu *

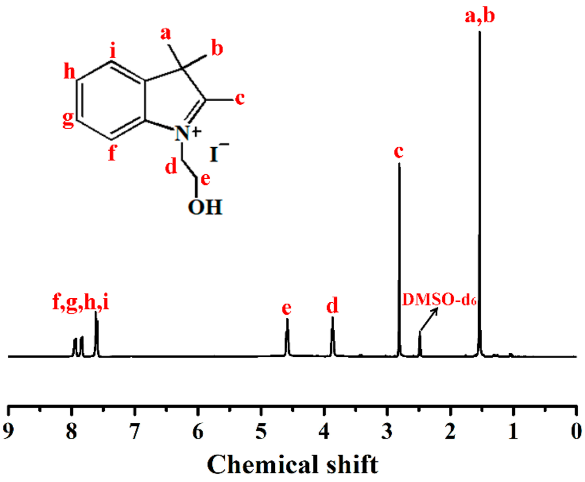

Figure S1. ^{1}H NMR spectrum of 2-hydroxyethyl-2,3,3-trimethyl-3H-indolium iodide.

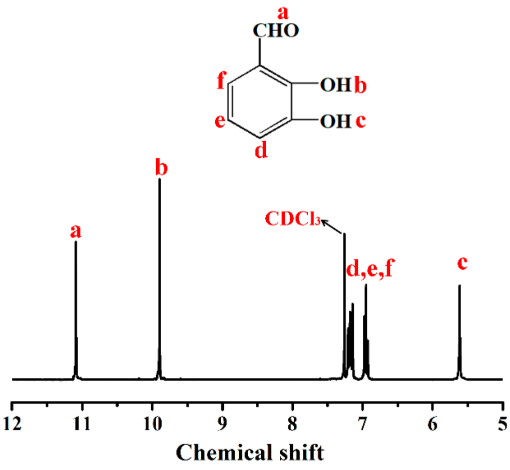

Figure S2. ^{1}H NMR spectrum of 2,3-dihydroxybenzaldehyde.

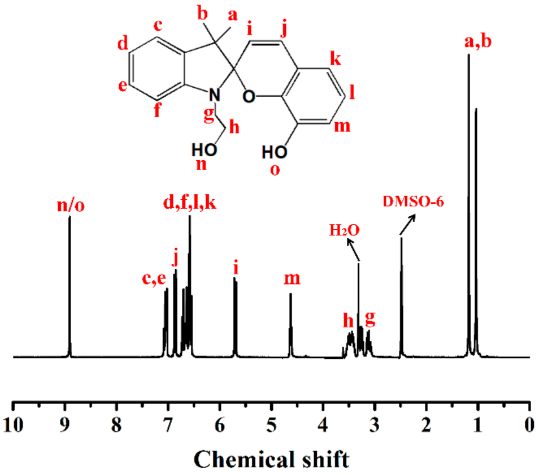

Figure S3. ^{1}H NMR spectrum of dihydroxyl spiropyran.

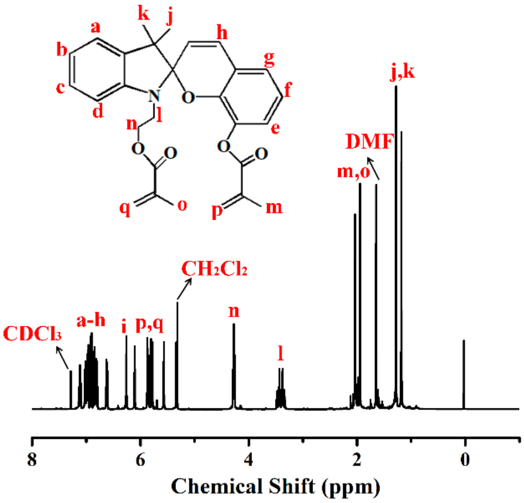

Figure S4. ^{1}H NMR spectrum of DMSP3 crosslinker.
